# Emotional and qualitative outcomes among patients with left and right hemisphere stroke

**DOI:** 10.3389/fneur.2022.969331

**Published:** 2022-11-17

**Authors:** Melissa D. Stockbridge, Emilia Vitti, Andreia V. Faria, Argye E. Hillis

**Affiliations:** ^1^Department of Neurology, Johns Hopkins University School of Medicine, Baltimore, MD, United States; ^2^Department of Radiology and Radiological Science, Johns Hopkins University School of Medicine, Baltimore, MD, United States

**Keywords:** stroke, depression, quality of life, lateralization, recovery, longitudinal

## Abstract

The differences in mental health outcomes of right and left hemisphere strokes are well studied; however, there is a long-standing controversy surrounding whether depression is associated with lateralization of stroke or not. In this investigation, we examined the effect of lesion location on post-stroke depression controlling for lesion size and hemiparesis in a longitudinal sample assessed at acute, subacute, and chronic timepoints. As a secondary aim, we further examined the effect of lesion location on self-reported difficulties across a wide array of domains. A series of 134 patients with left hemisphere strokes and 79 with right hemisphere strokes completed the Patient Health Questionnaire-9 and an inventory of post-stroke abilities at within acute, subacute, and chronic windows following stroke. When controlling for hemiparesis and overall lesion volume, we found no difference in depression between groups at any timepoint. Additional exploratory analyses provided a further look at differing challenges associated with depression in each group.

## Introduction

The differences between functional changes associated with right (RH) and left (LH) hemisphere strokes are numerous and well-studied. Differences in mental health and quality of life are less commonly examined with respect to lateralization yet are of particular interest to patients and their families who are trying to gain a more global perspective of what to expect in recovery.

Post-stroke depression impacts around one in three stroke survivors (1, 2) (about four times greater than the incidence of depression in the general population) and is an important mediator of the effects of rehabilitation on recovery. Depression is known to correlate with decreased serum brain-derived neurotrophic factor (BDNF) (3, 4), which is in turn associated with poorer stroke recovery (5–7), and may constitute a primary mechanism of this interaction (8). Recent work has highlighted the role depression plays in impeding recovery (9–11). It can lead to decreased rehabilitation therapy use (12) and poor post-stroke quality of life (13).

RH strokes have been more closely associated with the increased risk of depression according to one recent meta-analysis (14). However, there is a long-standing controversy surrounding whether depression is most common in those with left anterior and right posterior lesions (particularly the left frontal pole) and least common in those with left posterior and right anterior lesions, at least when examined the acute and subacute phase (15–19), or whether there is no such lateral association (20–24). Differences in methodology and ability to assess depression in individuals with language-disruption (aphasia) due to LH stroke perhaps have contributed to the mixed landscape of prior literature (25).

In this investigation, we examined the effect of lesion location on post-stroke depression controlling for lesion size and hemiparesis in a longitudinal sample assessed at acute, subacute, and chronic timepoints. As a secondary aim, we further examined the effect of lesion location on self-reported difficulties across a wide array of domains.

## Materials and methods

### Recruitment

A total of 231 participants, including 141 with an acute LH lesion and 90 with an acute RH lesion were identified through ongoing longitudinal protocols investigating stroke recovery. All participants were right-handed, native English speakers with normal or corrected-to-normal vision and hearing (participants and overall sample statistics are described in Table 1). In order to account for lesion volume, lesions were analyzed in MNI space (1 voxel = 1 mm^3^). All scans were visually inspected following the automated processes for quality control. In order to account for hemiparesis, the most acute NIH Stroke Scale administered during the intake neurological examination was examined. Individuals who scored points on items 5 (upper limb strength) or 6 (lower limb strength) were categorized as having hemiparesis. Patients with additional chronic lesions were included if the lesions were small and apparently asymptomatic. Individuals with bilateral acute lesions, as well as those with other underlying diagnoses impacting cognition and language (e.g., dementia) were excluded. Recovery periods were defined as acute (within the first 14 days), subacute (15–90 days), and chronic (over 90 days). When an individual was evaluated more than once in a given period, scores were averaged.

**Table 1:** Group description.

	**LH (*N* = 134)**	**RH (*N* = 79)**
Age	62.5 (12.1)	61.0 (15.0)
Sex (F:M)^*^	68:66	37:42
Education	14.6 (2.9)	14.4 (3.1)
**NIH SS**	3.4 (3.4)	4.4 (4.8)
Hemiparesis (Y:N)^*^	50:77	46:32
**PHQ-9 classification (count, %)**	N = 108	N = 70
None/Minimal	54 (50%)	35 (50%)
Mild	33 (31%)	22 (31%)
Moderate	13 (12%)	7 (10%)
Moderate-Severe	4 (4%)	5 (7%)
Severe	4 (4%)	1 (1%)
Intracranial volume (cc)	1,318.0 (188.4)	1,319.7 (183.5)
Lesion size (cc)^*^	14.1 (22.5)	27.4 (37.1)
L Frontal	3.9 (11.1)	0.3 (1.6)
L Parietal	4.3 (10.0)	0.3 (1.6)
L Temporal	3.5 (9.2)	0.4 (3.1)
L Occipital	2.5 (6.7)	0.1 (0.4)
R Frontal	0.3 (2.1)	9.9 (16.3)
R Parietal	0.1 (1.2)	7.7 (13.7)
R Temporal	0.0 (0.1)	5.4 (11.1)
R Occipital	0.4 (3.7)	1.7 (3.6)

* *p* < 0.05.

### Questionnaires

Two questionnaires were identified for analysis: Patient Health Questionnaire – 9 [PHQ; (26–28)] and the Stroke Patient Questionnaire (SPQ). The PHQ-9 is a widely-employed and psychometrically strong 9-item self-report tool ideal for capturing major depression after stroke (29–32). The SPQ is a 50-item self-reported inventory of challenges patients may experience in mobility, cognition, language, mood, activities of daily living, and participation in home management, social, and medical activities. Patients first identify whether each inventory item occurred in the past month with a “yes” or “no,” then rate “how difficult or concerning” the endorsed problems have been on a scale of 1 “Not at all” to 3 “A lot.” Due to the time constraints associated with testing in an acute setting, not all patients received all items. Items 1–3, 19, 21–29, 32, 33, 35, 37–39, 42–44, and 48–50 were removed from the questionnaire for a number of patients with LH damage. These items predominantly address mobility, loneliness, mood changes, activities of daily living, and participation in social and medical activities. Individuals with RH stroke also did not consistently receive items 1–3, 15, 17–19, 21–50, but two additional inventory items were included in these administrations: (1) “had others say he/she is monotone or that he/she can't fully express emotions to others” and (2) “cared that his/her voice is monotone or that he/she can't fully express emotions to others.” These items were included to reflect the prevalence of aprosodia (33) in RH stroke. The 50-item and RH SPQ are included in Supplementary materials. SPQ ratings were divided by the total number of items to arrive at a proportion of items endorsed and average rating that were used in analysis.

In order to arrive at the most reliable questionnaire responses possible for analysis, individuals who had received additional cognitive or linguistic testing at a given timepoint were excluded if they demonstrated poor accuracy when responding to tasks targeting comprehension of simple language (e.g., “Is your name Bob?”). In individuals with left hemisphere lesions, the task used to determine reliability was the Western Aphasia Battery Auditory Verbal Comprehension section (participants are asked to respond with “yes” or “no” to 60 questions about themselves and their surroundings). For individuals with right hemisphere lesions, emotion synonym matching was selected as the most frequently used measure that would capture comprehension. In this task, participants are asked to identify whether an emotion word is synonymous or not. When this was not available, an emotional semantics test was used instead. In both cases, individuals had to have achieved a score of 80% or higher on the appropriate measure in order for their questionnaire data to be analyzed (at least 48/60 points on the WAB Yes/No and 19/24 on the synonym matching). In most cases, administration of questionnaires had not been attempted on highly unreliable patients (i.e., there were no instances of questionnaires attempted on a person with global aphasia); however, a limited set of questionnaires were dropped due to not meeting this threshold. Acute data removed included 16 PHQ-9s (6 LH, 10 RH) and 14 SPQs (3 LH, 11 RH). Two subacute PHQ-9s and 1 SPQ were removed from the RH subacute data. From the chronic data, 3 PHQ-9s (2 LH, 1 RH) were removed. When this process was completed, 7 individuals with LH lesions and 11 individuals with RH lesions were removed due to having no remaining data to be analyzed, leaving 134 with acute left hemisphere lesions and 79 with acute right hemisphere lesions to be analyzed.

### Analysis

In order to examine the effect of lesion location on post-stroke depression controlling for lesion size and hemiparesis, a univariable analysis of variance was calculated with lesion hemisphere as the fixed factor and PHQ-9 total score at each timepoint as dependent variables, controlling for hemiparesis and lesion volume. This was then repeated for proportion of SPQ items endorsed and mean rating of endorsed SPQ items as dependent variables.

Two additional planned analyses were conducted to comprehensively describe the data. First, among those with LH damage, we examined whether acute aphasia type on the WAB was associated with depression when controlling for lesion volume and hemiparesis. This was done to better understand the relationship between the unique challenges associated with different aphasia types and post-stroke depression. Second, we examined whether SPQ item endorsement correlated with PHQ for either the RH or LH groups. This was done to better understand the relationship between more objective reported challenges (e.g., ability to drive or to bathe independently) and post stroke depression.

## Results

LH and RH groups were similar with regard to age [t_(138.2)_ = 0.79, *p* = 0.43], education [t_(201)_ = 0.47, *p* = 0.64], intracranial volume [t_(188)_ = 0.06, *p* = 0.95], baseline NIH SS [t_(125.4)_ = 1.65, *p* = 0.10]. However, in the RH group there was a higher proportion of men [$\chi_{\left(4\right)}^{2}$ = 253.36, *p* < 0.001], individuals with hemiparesis [$\chi_{\left(1\right)}^{2}$ = 7.46, *p* = 0.01] and larger lesions on average [t_(101.4)_ = 2.75, *p* = 0.01; see Table 1]. When available for those with LH lesions, acute WAB performance indicated that 31 did not have aphasia, 31 were anomic, 3 had Broca's aphasia, 3 had Conduction aphasia, 4 had Transcortical Motor aphasia, 1 had Transcortical sensory aphasia, and 1 had Wernicke's aphasia.

### Depression

PHQ-9 interpretation by lateralization is included in Table 1. There was no statistically significant difference in depression on the PHQ-9 based on lesion lateralization when controlling for hemiparesis and stroke volume at the acute, F_(1, 149)_ = 2.47, *p* = 0.75, subacute, F_(1, 37)_ = 0.48, *p* = 0.49, or chronic, F_(1, 94)_ = 0.21, *p* = 0.65 time points. When controlling for hemiparesis and stroke volume, lesion volume to each lobe did not correlate with depression measured at any time point (Table 2). There was no significant difference in depression as a function of aphasia type in our sample when controlling for hemiparesis and lesion volume, F_(7, 143)_ = 1.06, *p* = 0.40.

**Table 2:** Correlations between depression and lesion size by lobe.

	**Acute**	**Subacute**	**Chronic**
L Frontal	−0.12	0.06	−0.09
L Parietal	0.09	0.04	−0.1
L Temporal	−0.07	0.08	−0.13
L Occipital	−0.1	−0.04	0.04
R Frontal	0.33	0.1	0.09
R Parietal	0.11	−0.1	0.37
R Temporal	0.27	0.23	0.39
R Occipital	−0.06	−0.16	0.33

### Disability and quality of life

**Figure 1:**
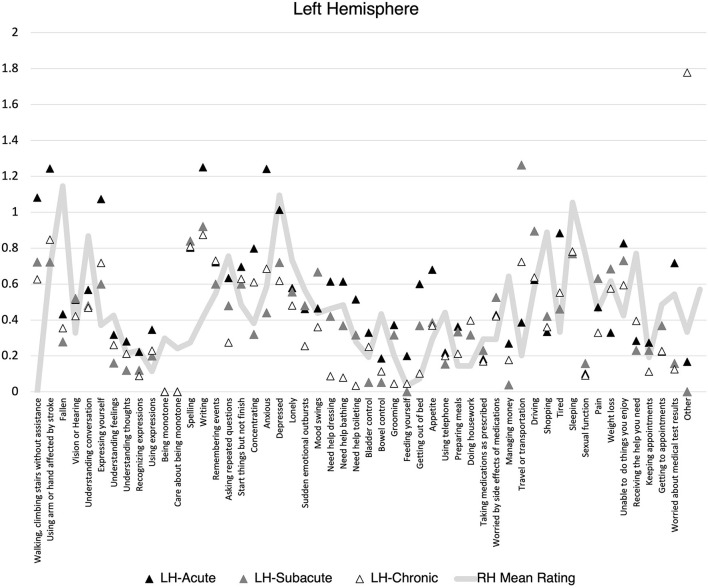
Mean Stroke Patient Questionnaire (SPQ) item rating (0-2) by recovery time point for individuals with left hemisphere stroke. Mean right hemisphere rating included for reference.

**Figure 2:**
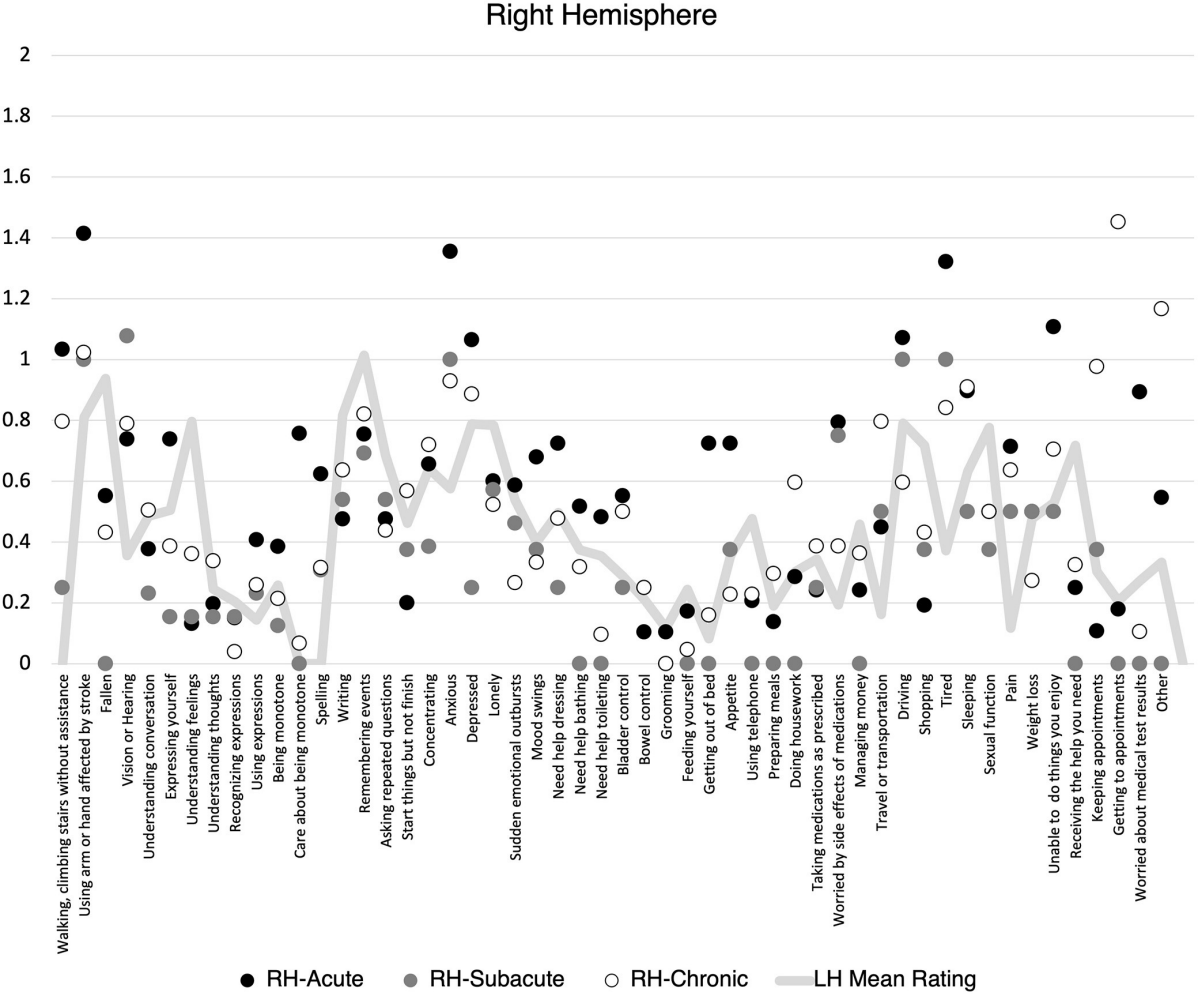
Mean Stroke Patient Questionnaire (SPQ) item rating (0-2) by recovery time point for individuals with right hemisphere stroke. Mean left hemisphere rating included for reference.

There was no statistically significant difference in the proportion of items endorsed on the SPQ based on lesion lateralization when controlling for hemiparesis and stroke volume at the acute, F_(1, 118)_ = 0.59, *p* = 0.44, subacute, F_(1, 31)_ = 0.06, *p* = 0.81, or chronic, F_(1, 88)_ = 0.01, *p* = 0.92 timepoints. When individuals did endorse an item, there was no difference mean severity rating at the acute, F_(1, 118)_ = 0.89, *p* = 0.35, subacute, F_(1, 31)_ = 0.04, *p* = 0.84, or chronic, F_(1, 88)_ = 0.34, *p* = 0.56, timepoints. However, patients clearly demonstrated different levels of endorsement at the item level depending on the lateralization of their lesion (Figures 1, 2). Correlations between PHQ-9 and SPQ items are included in Table 3.

**Table 3:** Exploratory correlations between stroke patient questionnaire items and depression.

	**Overall**			**Left**			**Right**		
	**A**	**S**	**C**	**A**	**S**	**C**	**A**	**S**	**C**
Walking, climbing stairs without assistance		0.76**			0.76**			0.94*	0.54*
Using arm or hand affected by stroke		0.43*	0.29*		0.67**				0.61*
Fallen		0.58**	0.38**		0.59*	0.41**			
Vision or Hearing	0.21*		0.34**			0.40**	0.41**		
Understanding conversation	0.31**	0.44**	0.29**	0.27*		0.35**	0.39**	0.70*	
Expressing yourself	0.18*	0.46**	0.39**			0.48**	0.26*		
Understanding feelings	0.21*	0.58**	0.24*		0.59**	0.29*	0.42**		
Understanding thoughts			0.37**			0.48**			
Recognizing expressions	0.21*	0.59**			0.62**		0.32*		
Using expressions	0.19*	0.53**	0.26*		0.54**				0.57**
Being monotone									
Care about being monotone									
Spelling	0.26**	0.36*	0.23*	0.31**		0.31*		0.79**	
Writing	0.22*	0.44**	0.33**			0.35**	0.30*		
Remembering events	0.40**	0.62**	0.55**	0.33**	0.66**	0.64**	0.51**	0.69*	0.42*
Asking repeated questions	0.24**	0.43*	0.40**			0.46**	0.31*	0.66*	
Start things but not finish	0.54**	0.43*	0.47**	0.51**		0.53**	0.70**	0.94*	
Concentrating	0.45**		0.53**	0.46**		0.60**	0.42**		0.43*
Anxious	0.35**		0.58**	0.38**		0.61**			0.59*
Depressed	0.38**	0.65**	0.61**	0.43**	0.64**	0.60**		0.94*	0.74**
Lonely	0.40**	0.49*	0.50**	0.50**		0.50**		0.94*	0.55*
Sudden emotional outbursts	0.43**	0.42*	0.43**	0.36**		0.40**	0.52**	0.65*	0.49**
Mood swings	0.37**	0.49*	0.29*	0.40**				0.94*	
Need help dressing									
Need help bathing									
Need help toileting									
Bladder control								0.94*	
Bowel control								0.94*	
Grooming			0.26*						
Feeding yourself	0.32**			0.33**					
Getting out of bed			0.30*						
Appetite	0.31**		0.40**	0.30**		0.44**		0.94*	
Using telephone			0.25*			0.30*			
Preparing meals									0.73**
Doing housework			0.35**			0.36*			
Taking medications as prescribed		0.58**	0.36**		0.63**	0.44**	0.50**	0.94*	
Worried by side effects of medications	0.23*							0.91*	
Managing money			0.38**			0.44**			
Travel or transportation		0.43*	0.26*		0.50*				0.52*
Driving									
Shopping			0.41**			0.44**			
Tired	0.49**		0.52**	0.52**		0.58**	0.54**		
Sleeping	0.27**	0.69**	0.60**	0.24*	0.75**	0.62**	0.40*		0.63*
Sexual function		0.57**			0.73**			0.94*	
Pain									
Weight loss				0.26*					
Unable to do things you enjoy	0.30**		0.34**	0.35**	0.42*	0.29*			0.57*
Receiving the help you need		0.53**			0.54**				
Keeping appointments	0.32**			0.32**	0.54**				
Getting to appointments		0.58**			0.58*	0.38*	0.41*		
Worried about medical test results	0.21*	0.69**		0.32*	0.72**				

^**^p = 0.01, ^*^p = 0.05, A, Acute; S, Subacute; C, Chronic. The cells are shaded to denote the levels of significance.

## Discussion

This study examined the relationship between lesion lateralization and post-stroke depression. Numerous studies have led to a landscape of mixed results with some studies supporting a link between RH and depression (14), some supporting a link between LH and depression (15–19), and some finding no reliable link between lateralization and depression at all (20–24). Our study included a number of methodological decisions that we feel controlled for additional factors that could explain the heterogeneity of findings in prior work. We controlled for overall lesion size and acute hemiparesis. We also utilized a larger sample than many prior studies and examined that sample at three timepoints: acute, subacute, and chronic. We also made a concerted effort to remove self-reported ratings from individuals whose other test scores suggested they were unlikely to fully comprehend the nuance in the items they were responding to. To our knowledge, this is the first study of its kind to account for comprehension deficits to this degree (i.e., often studies of RH do not include a task that would permit this, or the items examined are far easier and less nuanced than the items used in depression questionnaires). While doing this does limit the ability of individuals with severe language deficits to participate, for the purposes of examining the effect of lateralization specifically, it was an important quality assurance step. In doing so, our study affirmed the finding from Carson et al. (24) and others that there is no reliable difference in depression between those with RH and LH lesions, even when considered in the acute phase. Aphasia types also were associated with similar levels of depression.

Additional exploratory analyses sought to provide nuance to this finding by examining the relationship between depression and self-reported endorsement of common areas of difficulty patients face when recovering from stroke. Items targeted changes to mobility, cognition, language, activities of daily living, and participation in home management, social, and medical activities. The findings from this exploration were interesting and inspire many questions for future study. For people with both LH and RH lesions, lingering difficulties with mobility extending beyond 3 months, communication & social problems, exhaustion, and adapting to changes in medical management needs were common areas that were associated with increased depression. Perhaps unsurprisingly, for individuals with LH lesions, depression was most associated with communication & social problems throughout the year following stroke. There was some evidence of endorsed language problems at the acute phase with social challenges and changes to independence (e.g., money, housework) being more strongly associated with depression in subacute and chronic phases. This mirrors the common experience of many stroke survivors with aphasia who struggle with isolation and challenges to participation long after the acute challenges of their stroke have subsided (34, 35). Patients with LH lesions had a strong association between depression and the ability to do things they enjoyed and get help when needed. In contrast, individuals with RH demonstrated strong associations between depression and acute hearing, vision changes, and social communication difficulties, and ongoing cognitive challenges.

Perhaps as interesting was what didn't associate with depression in our sample. Patients did not seem to demonstrate strong relationships between depression and needing assistance with activities of daily living, losing the ability to drive, pain, or weight loss at any timepoint. This was an interesting finding, as these types of challenges while not cognitively taxing are often associated with a lack of functional independence and perceived burden on others (36–38). An important caveat of this exploratory work is that it does not presume to answer questions regarding the directionality of these relationships. It also does not address the extent to which post-stroke depression could drive exaggerated ratings of difficulty, though there is evidence to suggest this is likely (39, 40) and should be considered in future studies of depression and the various domains of stroke recovery.

## Data availability statement

The original contributions presented in the study are included in the article/Supplementary material, further inquiries can be directed to the corresponding author/s.

## Ethics statement

The studies involving human participants were reviewed and approved by Johns Hopkins University School of Medicine Institutional Review Board. The patients/participants provided their written informed consent to participate in this study.

## Author contributions

AH provided the funding. MS and AH conceived of the study. EV aggregated the data. AF and MS analyzed the data. MS wrote the initial draft and all authors edited the manuscript prior to submission. All authors contributed to the article and approved the submitted version.

## Funding

This work was supported by National Institutes of Health/National Institute on Deafness and Other Communication Disorders (NIH/NIDCD): P50 DC014664, R01 DC015466, and R01 DC05375.

## Conflict of interest

The authors declare that the research was conducted in the absence of any commercial or financial relationships that could be construed as a potential conflict of interest.

## Publisher's note

All claims expressed in this article are solely those of the authors and do not necessarily represent those of their affiliated organizations, or those of the publisher, the editors and the reviewers. Any product that may be evaluated in this article, or claim that may be made by its manufacturer, is not guaranteed or endorsed by the publisher.
