# Supplementary material for: Emotional and qualitative outcomes among patients with left and right hemisphere stroke
Source: Front Neurol. 2022 Nov 17;13:969331. doi: 10.3389/fneur.2022.969331 (PMC9712731; doi:10.3389/fneur.2022.969331)
Supplement: Supplementary file 2 [file Data_Sheet_1.PDF]

Pt Code: ____________ Date: ____________ Study time point: ____________ DOB: _____________

Stroke Patient Questionnaire - Hopkins

Stroke can cause many problems. Here is a list of problems that you may have experienced since your stroke, or other problems that may be disrupting your daily life. Please indicate if any of these problems has occurred within the past month. If it has occurred, indicate how difficult this problem is for you and how concerned you are about the problem on a 3-point scale:

1=Not at all 2= A little 3=A lot

In the past month, have you…………	Did this problem occur in the past month?		If Yes, how difficult or concerning is this problem for you?		
1. had difficulty walking or climbing stairs without assistance	Yes	No	1	2	3
2. had problems using the arm or hand that was most affected by the stroke	Yes	No	1	2	3
3. fallen	Yes	No	1	2	3
4. had problems with vision or hearing	Yes	No	1	2	3
5. difficulty understanding what was said to you in conversations	Yes	No	1	2	3
6. had difficulty expressing yourself or participating in conversations	Yes	No	1	2	3
7. had trouble understanding the feelings of others	Yes	No	1	2	3
8. had trouble understanding the thoughts of others	Yes	No	1	2	3
9. had trouble recognizing the tone of voice and facial expressions of others	Yes	No	1	2	3
10. had trouble using tone of voice and facial expression to show emotion	Yes	No	1	2	3
11. had problems with spelling	Yes	No	1	2	3
12. had problems with writing	Yes	No	1	2	3
13. had trouble remembering recent events	Yes	No	1	2	3
14. been asking the same question over and over	Yes	No	1	2	3
15. been starting, but not finishing things	Yes	No	1	2	3
16. had difficulty concentrating on a task	Yes	No	1	2	3
17. felt anxious or worried	Yes	No	1	2	3
18. felt sad or depressed	Yes	No	1	2	3
19. Felt lonely	Yes	No	1	2	3
10. had sudden outbursts of uncontrollable crying or laughing	Yes	No	1	2	3
21. had mood swings	Yes	No	1	2	3

Pt Code: ____________ Date: ____________ Study time point: ____________ DOB: _____________

In the past month, have you…………	Did this problem occur in the past month?		If Yes, how difficult or concerning is this problem for you?		
22. needed help with dressing	Yes	No	1	2	3
23. needed help with bathing	Yes	No	1	2	3
24. needed help with toileting	Yes	No	1	2	3
25. had a problem controlling your bladder	Yes	No	1	2	3
26. had a problem controlling your bowels	Yes	No	1	2	3
27. needed help with grooming such as brushing teeth or combing hair	Yes	No	1	2	3
28. needed help with eating or feeding yourself	Yes	No	1	2	3
29. needed help with getting in and out of bed	Yes	No	1	2	3
30. had a decrease in appetite	Yes	No	1	2	3
31. needed help using the telephone	Yes	No	1	2	3
32. needed help preparing meals	Yes	No	1	2	3
33. needed help doing housework	Yes	No	1	2	3
34. needed help taking your medications as prescribed	Yes	No	1	2	3
35. been worried by the side effects of medications	Yes	No	1	2	3
36. needed help managing your money	Yes	No	1	2	3
37. needed help with travel or transportation	Yes	No	1	2	3
38. had concerns about driving	Yes	No	1	2	3
39. needed help with shopping	Yes	No	1	2	3
40. been more tired than usual	Yes	No	1	2	3
41. had difficulty sleeping	Yes	No	1	2	3
42. had difficulty with sexual function	Yes	No	1	2	3
43. been in pain	Yes	No	1	2	3
44. lost weight	Yes	No	1	2	3
45. been unable to get out to do the things you enjoy	Yes	No	1	2	3
46. had difficulty receiving the help you need	Yes	No	1	2	3
47. had difficulty keeping up with appointments	Yes	No	1	2	3
48. had difficulty getting to appointments	Yes	No	1	2	3
49. been worried by the results of a lab test or consultation	Yes	No	1	2	3
50. other: ____________________________	Yes	No	1	2	3

Stroke Patient Questionnaire Total ______________________
