# Supplementary material for: Emotional and qualitative outcomes among patients with left and right hemisphere stroke
Source: Front Neurol. 2022 Nov 17;13:969331. doi: 10.3389/fneur.2022.969331 (PMC9712731; doi:10.3389/fneur.2022.969331)
Supplement: Supplementary file 3 [file Data_Sheet_2.PDF]

Date:________________ Participant ID:______________ Relation to Participant:________________

Stroke Patient Companion Questionnaire

Stroke can cause many problems. Here is a list of problems that your family member or friend may have experienced since his/her stroke, or other problems that may be disrupting that person's daily life. Please indicate if your family member or friend has had any of these problems within the past month. It a problem has occurred, indicate whether you provide any help with that problem. Use the 3-point scale to indicate how difficult or upsetting it is for you to provide that help. **1 = Not at all, 2 = A little, 3 = A lot**

In the past month, has your family member/friend…	Did this problem occur in the past month?		Did you provide any help with this problem?		If yes, how difficult or upsetting was it for you to provide that help?		
1. had problems with vision or hearing	Yes	No	Yes	No	1	2	3
2. had difficulty understanding what was said to them in conversations	Yes	No	Yes	No	1	2	3
3. had difficulty expressing themselves or participating in conversations	Yes	No	Yes	No	1	2	3
4. had trouble understanding the feeling of others	Yes	No	Yes	No	1	2	3
5. had trouble understanding the thoughts of others	Yes	No	Yes	No	1	2	3
6. had trouble recognizing the tone of voice and facial expressions of others	Yes	No	Yes	No	1	2	3
7. had trouble using tone of voice and facial expression to show emotion	Yes	No	Yes	No	1	2	3
8. had others say he/she is monotone or that he/she can't fully express emotions to others	Yes	No	Yes	No	1	2	3
9. cared that his/her voice is monotone or that he/she can't fully express emotions to others	Yes	No	Yes	No	1	2	3
10. had problems with spelling	Yes	No	Yes	No	1	2	3
11. had problems with writing	Yes	No	Yes	No	1	2	3
12. had trouble remembering recent events	Yes	No	Yes	No	1	2	3
13. been asking the same question(s) over and over	Yes	No	Yes	No	1	2	3
14. had difficulty concentrating on a task	Yes	No	Yes	No	1	2	3
15. had sudden outbursts of uncontrollable crying or laughing	Yes	No	Yes	No	1	2	3
16. Other:________________________	Yes	No	Yes	No	1	2	3

Score:__________
